# *catena*-Poly[[[diaqua­nickel(II)]-μ-pyrazine-2-carboxyl­ato-silver(I)-μ-pyrazine-2-carboxyl­ato] nitrate dihydrate]

**DOI:** 10.1107/S1600536812018119

**Published:** 2012-04-28

**Authors:** Min Yang, Li-Yuan Chai, Xiao-Yi Yi

**Affiliations:** a Key Laboratory of Resources Chemistry of Nonferrous Metals, Ministry of Education, Central South University, Changsha, Hunan Province 410083, People’s Republic of China; b Institute of Environmental Engineering, Central South University, Changsha, Hunan Province 410083, People’s Republic of China

## Abstract

In the polymeric complex of the title compound, {[AgNi(C_5_H_3_N_2_O_2_)_2_(H_2_O)_2_]NO_3_·2H_2_O}_*n*_, the Ag^I^ ion displays an angular coordination geometry with two N atoms from pyrazine-2-carboxyl­ate ligands, and the Ni^II^ ion is hexa­coordinated by two O atoms from two water mol­ecules, two O and two N atoms from pyrazine-2-carboxyl­ate ligands in a distorted octa­hedral geometry. In the crystal, the Ag^I^ and Ni^II^ ions lie on a mirror plane and an inversion centre, respectively. The complex chains, the nitrate ions and the uncoordinated water mol­ecules are linked together through O—H⋯O hydrogen bonds and weak Ag⋯O inter­actions [2.619 (17)–2.749 (17) Å] into a three-dimensional network.

## Related literature

A similar one-dimensional chain mixed-metal Co–Ag coord­ination polymer {[AgCo(C_4_H_3_N_2_CO_2_)_2_(H_2_O)]NO_3_}_*n*_ (Ciurtin *et al.*, 2002 ▶) and a pillared Ni–Ag–Re polymer {[AgNi(C_4_H_3_N_2_CO_2_)_2_(H_2_O)_2_](ReO_4_)}_*n*_ (Maggard *et al.*, 2005 ▶) have been reported.
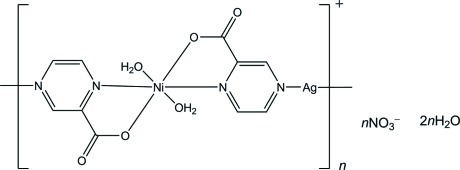


## Experimental

### 

#### Crystal data

[AgNi(C_5_H_3_N_2_O_2_)_2_(H_2_O)_2_]NO_3_·2H_2_O
*M* *_r_* = 546.84
Monoclinic, 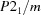

*a* = 5.1997 (10) Å
*b* = 27.188 (5) Å
*c* = 6.4347 (13) Å
β = 111.24 (3)°
*V* = 847.9 (3) Å^3^
*Z* = 2
Mo *K*α radiation
μ = 2.34 mm^−1^
*T* = 293 K
0.15 × 0.10 × 0.08 mm

#### Data collection

Rigaku Mercury diffractometer
Absorption correction: multi-scan (*CrystalClear*; Rigaku, 2005 ▶) *T*_min_ = 0.61, *T*_max_ = 0.98
8224 measured reflections
1972 independent reflections
1526 reflections with *I* > 2σ(*I*)
*R*_int_ = 0.065

#### Refinement

*R*[*F* ^2^ > 2σ(*F* ^2^)] = 0.070
*wR*(*F* ^2^) = 0.245
*S* = 1.01
1972 reflections
136 parameters
H-atom parameters constrained
Δρ_max_ = 1.31 e Å^−3^
Δρ_min_ = −1.09 e Å^−3^

### 

Data collection: *CrystalClear* (Rigaku, 2005 ▶); cell refinement: *CrystalClear*; data reduction: *CrystalClear*; program(s) used to solve structure: *SHELXS97* (Sheldrick, 2008 ▶); program(s) used to refine structure: *SHELXL97* (Sheldrick, 2008 ▶); molecular graphics: *DIAMOND* (Brandenburg, 2005 ▶); software used to prepare material for publication: *SHELXL97*.

## Supplementary Material

Crystal structure: contains datablock(s) global, I. DOI: 10.1107/S1600536812018119/is5099sup1.cif

Structure factors: contains datablock(s) I. DOI: 10.1107/S1600536812018119/is5099Isup2.hkl

Additional supplementary materials:  crystallographic information; 3D view; checkCIF report

## Figures and Tables

**Table 1:** Hydrogen-bond geometry (Å, °)

*D*—H⋯*A*	*D*—H	H⋯*A*	*D*⋯*A*	*D*—H⋯*A*
O1*W*—H1*WA*⋯O1^i^	0.85	1.90	2.729 (7)	164
O1*W*—H1*WB*⋯O2^ii^	0.85	1.91	2.699 (7)	155
O2*W*—H2*WA*⋯O2^i^	0.85	2.09	2.926 (11)	166
O2*W*—H2*WB*⋯O4	0.85	2.11	2.883 (12)	151

Symmetry codes: (i) 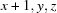; (ii) 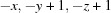.

**Fig. 1.**
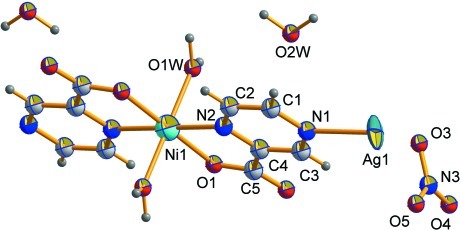
A polymeric one-dimensional chain showing 50% probability displacement ellipsoids.

**Fig. 2.**
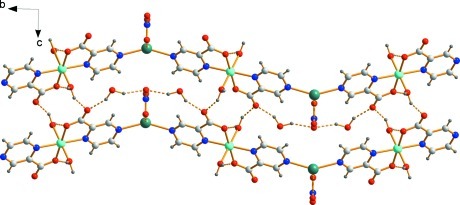
View of a two-dimensional network generated by O—H···O hydrogen bonding.

**Fig. 3.**
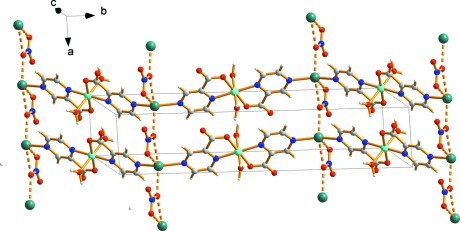
View of a two-dimensional network generated by Ag···O weak interactions.

## supplementary crystallographic information

### Comment

One of the major goals in inorganic chemistry is the self-assembly of polynuclear coordination arrays. The pyrazine-2-carboxylate (pyzc), as a bidentate herteroaromatic linker, is a suitable ligand to generate well defined architectures in a controlled fashion. Many mixed metal complexes with pyzc ligand have been reported. Here we describe the crystal structure of Ni–Ag coordination polymer {[AgNi(C_4_H_3_N_2_CO_2_)_2_(H_2_O)_2_)]NO_3_.2H_2_O}_n_.

The title complex is a polymeric structure consisting of Ni(C_4_H_3_N_2_CO_2_)_2_(H_2_O)_2_ units linked into infinite chains by Ag^+^ center [Ag1—N1 2.255 (7) Å] (Fig. 1). The pseudo-octahedral {NiO_4_N_2_} coordination environment around each Ni center consists of two O atoms from coordinated waters and two O and two N atoms from two chelating pyzc ligands [Ni1—O1 2.059 (5), Ni1—N2 2.079 (6), Ni1—O1w 2.057 (5) Å]. The hydrogen bonds are observed between coordinated water O1w and carboxylato O atom of pyzc ligand, and between uncoordinated water O2w and one carboxylato oxygen atom and one nitrito O atom (Table 1 and Fig. 2). The charge-balanced anionic nitrate ion acts both as a bidentate donor through O3 and O5 atoms [Ag—O3 2.749 (17), Ag—O5 2.712 (18) Å] and as a monodentate donor through O3 [Ag—O3 2.619 (17) Å] to be weakly bound to two Ag^+^ from two neighbor chain (Fig. 3). The combination of hydrogen bonding and weak Ag···O interactions serves to effectively link individual chains into a three-dimensional network.

### Experimental

A mixture of [Ni(pyzc)_2_(H_2_O)_2_].xH_2_O (17.0 mg, 0.05 mmol) and AgNO_3_ (8.5 mg, 0.05 mmol) in water (2 ml) was heated to 80 °C for 30 min. The resulting solution held there undisturbedly overnight. The darkish green block crystals suitable for the X-ray diffraction study were obtained (yield 10%).

### Refinement

H atoms were placed in geometrically idealized positions (C—H = 0.93 and O—H = 0.85 Å) and constrained to ride on their parents atoms with *U*_iso_(H) = 1.2*U*_eq_(C,O). The highest residual electron peak is located 1.12 Å from atom Ag and the deepest hole is 0.48 Å from atom O4. The most disagreeable reflections with Delta(F^2^)/e.s.d. > 8 have been omitted.

### Crystal data

**Table:**

[AgNi(C_5_H_3_N_2_O_2_)_2_(H_2_O)_2_]NO_3_·2H_2_O	*F*(000) = 544
*M**_r_* = 546.84	*D*_x_ = 2.142 Mg m^−^^3^
Monoclinic, *P*2_1_/*m*	Mo *K*α radiation, λ = 0.71073 Å
*a* = 5.1997 (10) Å	Cell parameters from 6237 reflections
*b* = 27.188 (5) Å	θ = 3.0–25.0°
*c* = 6.4347 (13) Å	µ = 2.34 mm^−^^1^
β = 111.24 (3)°	*T* = 293 K
*V* = 847.9 (3) Å^3^	Block, dark-green
*Z* = 2	0.15 × 0.10 × 0.08 mm

### Data collection

**Table:**

Rigaku Mercury diffractometer	1972 independent reflections
Radiation source: fine-focus sealed tube	1526 reflections with *I* > 2σ(*I*)
Graphite monochromator	*R*_int_ = 0.065
ω scans	θ_max_ = 27.5°, θ_min_ = 3.0°
Absorption correction: multi-scan (*CrystalClear*; Rigaku, 2005)	*h* = −6→6
*T*_min_ = 0.61, *T*_max_ = 0.98	*k* = −34→35
8224 measured reflections	*l* = −8→8

### Refinement

**Table:**

Refinement on *F*^2^	Primary atom site location: structure-invariant direct methods
Least-squares matrix: full	Secondary atom site location: difference Fourier map
*R*[*F*^2^ > 2σ(*F*^2^)] = 0.070	Hydrogen site location: inferred from neighbouring sites
*wR*(*F*^2^) = 0.245	H-atom parameters constrained
*S* = 1.01	*w* = 1/[σ^2^(*F*_o_^2^) + (0.180*P*)^2^] where *P* = (*F*_o_^2^ + 2*F*_c_^2^)/3
1972 reflections	(Δ/σ)_max_ < 0.001
136 parameters	Δρ_max_ = 1.31 e Å^−^^3^
0 restraints	Δρ_min_ = −1.09 e Å^−^^3^

### Special details

**Table:**

Experimental. IR (KBr, cm^-1^): 445(*m*), 478(*m*), 731(*m*), 791(*m*), 791(*m*), 872(*m*), 1050(*s*), 1160(*s*), 1380(*s*) [ν(N=O)], 1421(*m*), 1588(*m*), 1661(*s*) [ν(C=O)].
Geometry. All e.s.d.'s (except the e.s.d. in the dihedral angle between two l.s. planes) are estimated using the full covariance matrix. The cell e.s.d.'s are taken into account individually in the estimation of e.s.d.'s in distances, angles and torsion angles; correlations between e.s.d.'s in cell parameters are only used when they are defined by crystal symmetry. An approximate (isotropic) treatment of cell e.s.d.'s is used for estimating e.s.d.'s involving l.s. planes.
Refinement. Refinement of *F*^2^ against ALL reflections. The weighted *R*-factor *wR* and goodness of fit *S* are based on *F*^2^, conventional *R*-factors *R* are based on *F*, with *F* set to zero for negative *F*^2^. The threshold expression of *F*^2^ > σ(*F*^2^) is used only for calculating *R*-factors(gt) *etc*. and is not relevant to the choice of reflections for refinement. *R*-factors based on *F*^2^ are statistically about twice as large as those based on *F*, and *R*- factors based on ALL data will be even larger.

### Fractional atomic coordinates and isotropic or equivalent isotropic displacement parameters (Å^2^)

**Table:**

	*x*	*y*	*z*	*U*_iso_*/*U*_eq_	
Ag1	0.3011 (3)	0.2500	0.2938 (2)	0.0617 (5)	
Ni1	0.0000	0.5000	0.0000	0.0221 (4)	
N1	0.2323 (14)	0.3299 (2)	0.1956 (11)	0.0377 (15)	
N2	0.1250 (11)	0.4272 (2)	0.0665 (9)	0.0249 (12)	
N3	0.958 (4)	0.2500	0.613 (4)	0.083 (5)	
C1	0.3158 (15)	0.3494 (3)	0.0409 (14)	0.0358 (17)	
H1	0.4108	0.3299	−0.0261	0.043*	
C2	0.2639 (14)	0.3976 (3)	−0.0208 (12)	0.0278 (14)	
H2	0.3278	0.4102	−0.1276	0.033*	
C3	0.0911 (16)	0.3592 (3)	0.2839 (12)	0.0343 (16)	
H3	0.0267	0.3464	0.3900	0.041*	
C4	0.0390 (14)	0.4078 (2)	0.2212 (10)	0.0229 (13)	
C5	−0.1318 (13)	0.4409 (2)	0.3121 (10)	0.0231 (13)	
O1	−0.1725 (10)	0.48437 (18)	0.2343 (8)	0.0280 (11)	
O2	−0.2190 (12)	0.4236 (2)	0.4499 (9)	0.0370 (12)	
O3	0.838 (3)	0.2500	0.405 (3)	0.106 (5)	
O4	0.822 (4)	0.2500	0.728 (4)	0.130 (7)	
O5	1.220 (3)	0.2500	0.687 (3)	0.115 (6)	
O1W	0.3534 (10)	0.5258 (2)	0.2428 (8)	0.0377 (13)	
H1WA	0.5129	0.5184	0.2445	0.045*	
H1WB	0.3385	0.5345	0.3648	0.045*	
O2W	0.638 (2)	0.3502 (4)	0.7228 (15)	0.086 (3)	
H2WA	0.6676	0.3681	0.6253	0.104*	
H2WB	0.6292	0.3193	0.6999	0.104*	

### Atomic displacement parameters (Å^2^)

**Table:**

	*U*^11^	*U*^22^	*U*^33^	*U*^12^	*U*^13^	*U*^23^
Ag1	0.0824 (9)	0.0176 (5)	0.1053 (10)	0.000	0.0582 (8)	0.000
Ni1	0.0277 (7)	0.0187 (6)	0.0239 (6)	0.0046 (4)	0.0143 (5)	0.0031 (4)
N1	0.052 (4)	0.023 (3)	0.049 (4)	0.003 (3)	0.030 (3)	0.002 (3)
N2	0.025 (3)	0.023 (3)	0.029 (3)	0.008 (2)	0.014 (2)	0.006 (2)
N3	0.099 (12)	0.028 (6)	0.157 (17)	0.000	0.090 (13)	0.000
C1	0.038 (4)	0.022 (3)	0.053 (4)	0.009 (3)	0.024 (4)	0.001 (3)
C2	0.029 (3)	0.024 (3)	0.035 (4)	0.005 (3)	0.016 (3)	0.003 (3)
C3	0.050 (4)	0.021 (3)	0.037 (4)	0.006 (3)	0.021 (3)	0.005 (3)
C4	0.030 (3)	0.017 (3)	0.023 (3)	−0.003 (2)	0.011 (3)	−0.001 (2)
C5	0.025 (3)	0.022 (3)	0.021 (3)	0.003 (2)	0.007 (2)	−0.004 (2)
O1	0.031 (2)	0.027 (2)	0.031 (2)	0.010 (2)	0.018 (2)	0.0028 (19)
O2	0.054 (3)	0.033 (3)	0.034 (3)	0.002 (2)	0.029 (3)	0.001 (2)
O3	0.072 (9)	0.128 (15)	0.128 (13)	0.000	0.051 (9)	0.000
O4	0.160 (15)	0.073 (10)	0.23 (2)	0.000	0.164 (16)	0.000
O5	0.069 (9)	0.178 (19)	0.098 (10)	0.000	0.028 (8)	0.000
O1W	0.032 (3)	0.050 (3)	0.034 (3)	0.007 (2)	0.016 (2)	−0.011 (2)
O2W	0.124 (7)	0.065 (6)	0.092 (6)	−0.003 (5)	0.064 (6)	0.001 (4)

### Geometric parameters (Å, º)

**Table:**

Ag1—N1^i^	2.255 (7)	N3—O5	1.27 (2)
Ag1—N1	2.255 (7)	C1—C2	1.369 (10)
Ni1—O1W^ii^	2.057 (5)	C1—H1	0.9300
Ni1—O1W	2.057 (5)	C2—H2	0.9300
Ni1—O1	2.059 (5)	C3—C4	1.381 (10)
Ni1—O1^ii^	2.059 (5)	C3—H3	0.9300
Ni1—N2^ii^	2.079 (6)	C4—C5	1.521 (9)
Ni1—N2	2.079 (6)	C5—O2	1.226 (8)
N1—C1	1.331 (10)	C5—O1	1.272 (8)
N1—C3	1.340 (9)	O1W—H1WA	0.8500
N2—C2	1.333 (9)	O1W—H1WB	0.8500
N2—C4	1.339 (8)	O2W—H2WA	0.8501
N3—O4	1.194 (19)	O2W—H2WB	0.8501
N3—O3	1.26 (2)		
			
N1^i^—Ag1—N1	149.0 (4)	O4—N3—O5	124 (2)
O1W^ii^—Ni1—O1W	180.0 (3)	O3—N3—O5	117.0 (16)
O1W^ii^—Ni1—O1	88.8 (2)	N1—C1—C2	121.0 (7)
O1W—Ni1—O1	91.2 (2)	N1—C1—H1	119.5
O1W^ii^—Ni1—O1^ii^	91.2 (2)	C2—C1—H1	119.5
O1W—Ni1—O1^ii^	88.8 (2)	N2—C2—C1	122.4 (7)
O1—Ni1—O1^ii^	180.0 (3)	N2—C2—H2	118.8
O1W^ii^—Ni1—N2^ii^	92.4 (2)	C1—C2—H2	118.8
O1W—Ni1—N2^ii^	87.6 (2)	N1—C3—C4	121.6 (7)
O1—Ni1—N2^ii^	99.2 (2)	N1—C3—H3	119.2
O1^ii^—Ni1—N2^ii^	80.8 (2)	C4—C3—H3	119.2
O1W^ii^—Ni1—N2	87.6 (2)	N2—C4—C3	120.8 (6)
O1W—Ni1—N2	92.4 (2)	N2—C4—C5	116.9 (6)
O1—Ni1—N2	80.8 (2)	C3—C4—C5	122.2 (6)
O1^ii^—Ni1—N2	99.2 (2)	O2—C5—O1	126.0 (6)
N2^ii^—Ni1—N2	180.000 (1)	O2—C5—C4	118.2 (6)
C1—N1—C3	117.3 (6)	O1—C5—C4	115.8 (5)
C1—N1—Ag1	122.1 (5)	C5—O1—Ni1	115.2 (4)
C3—N1—Ag1	120.6 (5)	Ni1—O1W—H1WA	121.9
C2—N2—C4	117.0 (6)	Ni1—O1W—H1WB	116.2
C2—N2—Ni1	131.6 (5)	H1WA—O1W—H1WB	118.0
C4—N2—Ni1	111.4 (4)	H2WA—O2W—H2WB	116.8
O4—N3—O3	119 (2)		

Symmetry codes: (i) *x*, −*y*+1/2, *z*; (ii) −*x*, −*y*+1, −*z*.

### Hydrogen-bond geometry (Å, º)

**Table:**

*D*—H···*A*	*D*—H	H···*A*	*D*···*A*	*D*—H···*A*
O1*W*—H1*WA*···O1^iii^	0.85	1.90	2.729 (7)	164
O1*W*—H1*WB*···O2^iv^	0.85	1.91	2.699 (7)	155
O2*W*—H2*WA*···O2^iii^	0.85	2.09	2.926 (11)	166
O2*W*—H2*WB*···O4	0.85	2.11	2.883 (12)	151

Symmetry codes: (iii) *x*+1, *y*, *z*; (iv) −*x*, −*y*+1, −*z*+1.
